# Primary tuberculous osteomyelitis of the maxilla mimicking a refractory post-endodontic infection: A diagnostic challenge

**DOI:** 10.1016/j.idcr.2026.e02753

**Published:** 2026-09-10

**Authors:** Ashir KR, Manoj Vengal, Hamesh Gangadharan, P.K. Rajeesh Mohammed, A.P. Vipindas, S. Aswathi

**Affiliations:** a Department of Oral Medicine and Radiology, KMCT Dental College, Manassery, Kozhikode, Kerala, 673602, India; b Department of Oral Pathology, KMCT Dental College, Manassery, Kozhikode, Kerala, 673602, India; c Department of Oral and Maxillofacial Surgery, KMCT Dental College, Manassery, Kozhikode, Kerala, 673602, India

**Keywords:** Tubercular osteomyelitis, Extrapulmonary tuberculosis, Maxillary osteomyelitis, Moth-eaten appearance, Molecular diagnostics, Giant cell lesions

## Abstract

Primary extrapulmonary tuberculosis involving the maxillofacial region is rare and often poses a diagnostic challenge because of its atypical presentation and the absence of pulmonary involvement. We report a case of a 38-year-old woman who developed rapidly progressive osteomyelitis of the maxilla following elective root canal treatment. The lesion was unresponsive to empirical antibiotic therapy and standard dental intervention. Cone-beam computed tomography revealed extensive osteolytic destruction, and histopathological examination demonstrated granulomatous inflammation with Langhans giant cells. Systemic evaluation showed no evidence of pulmonary tuberculosis, with negative chest radiography, acid-fast bacillus smear of sputum, and Mantoux test results. Cartridge-based nucleic acid amplification testing (CBNAAT) of the curetted bone tissue detected Mycobacterium tuberculosis without rifampicin resistance, confirming the diagnosis. The patient responded favorably to surgical debridement, followed by standard anti-tubercular therapy. This case highlights the importance of considering primary tuberculous osteomyelitis in persistent, non-healing maxillary lesions, even in the absence of pulmonary disease, and underscores the value of combining clinicoradiological, histopathological, and molecular investigations for a timely diagnosis.

## Highlights

• Primary maxillary tuberculosis mimicked a refractory post-endodontic infection.
• CBCT demonstrated extensive osteolysis with cortical and sinus floor perforation.
• Histopathology and CBNAAT established the diagnosis of extrapulmonary TB.
• Early anti-tubercular therapy resulted in complete clinical recovery.
• Tuberculosis should be considered in persistent, non-healing maxillary lesions.

## Introduction

Despite advances in diagnosis and treatment, tuberculosis (TB), a mycobacterial infection with worldwide prevalence, remains a major global health concern. Although pulmonary tuberculosis is the most common form, extrapulmonary tuberculosis (EPTB) accounts for a significant proportion of cases and may occur in the absence of active pulmonary disease [1].

Skeletal tuberculosis is an uncommon manifestation of EPTB, and tuberculous osteomyelitis of the jaws is exceptionally rare, particularly involving the maxilla. Owing to its nonspecific clinical presentation and frequent absence of constitutional symptoms, maxillary tuberculosis often mimics odontogenic infections or other inflammatory conditions, resulting in delayed diagnosis and inappropriate treatment.

We report a rare case of primary tuberculous osteomyelitis of the maxilla that presented as a refractory post-endodontic infection in an immunocompetent patient without evidence of pulmonary tuberculosis infection. This case highlights the diagnostic challenges posed by this uncommon entity, underscoring the need to consider TB in the differential diagnosis of persistent, non-healing maxillary lesions that fail to respond to conventional dental treatment.

## Case report

A 38-year-old woman underwent elective single-visit root canal treatment for deep caries with asymptomatic pulpitis involving the maxillary left first and second premolars (teeth 24 and 25). On postoperative day 1, she developed severe pain that was managed with analgesics and anti-inflammatory medications. Over the subsequent two and a half weeks, the pain persisted intermittently, with episodes of exacerbation and partial relief despite medication.

**Fig. 1:**
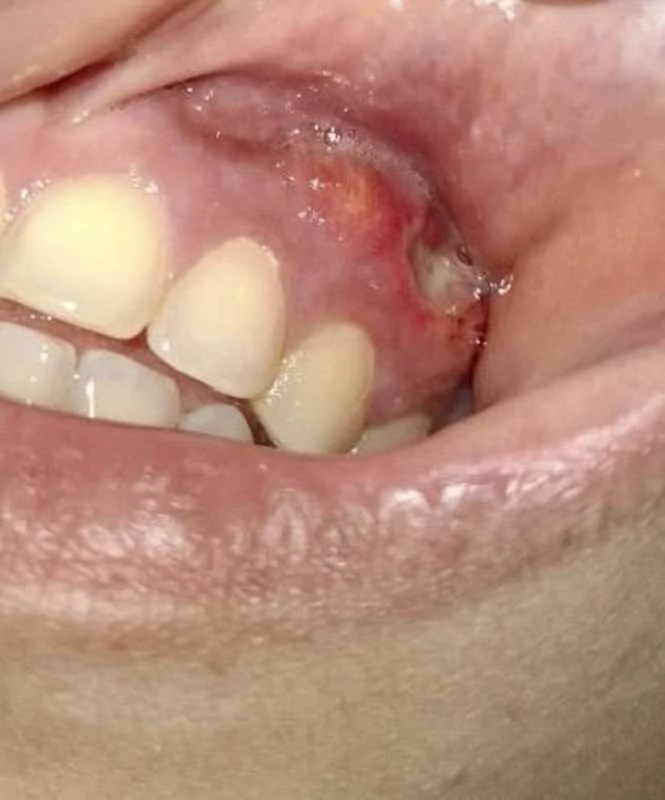
Multiple purulent sinus openings on the attached gingiva adjacent to and mesial to tooth 24.

By postoperative day 19, the patient developed swelling in the affected region, along with multiple purulent sinus openings on the attached gingiva adjacent to and mesial to tooth 24 (Fig. 1). Root canal retreatment of tooth 24 was initiated by removing the obturation material and irrigation of the canal, followed by empirical antibiotic therapy with amoxicillin–clavulanic acid (625 mg, orally, thrice daily) and metronidazole (400 mg, orally, thrice daily), both prescribed for 5 days. However, no clinical improvements were observed.

**Fig. 2:**
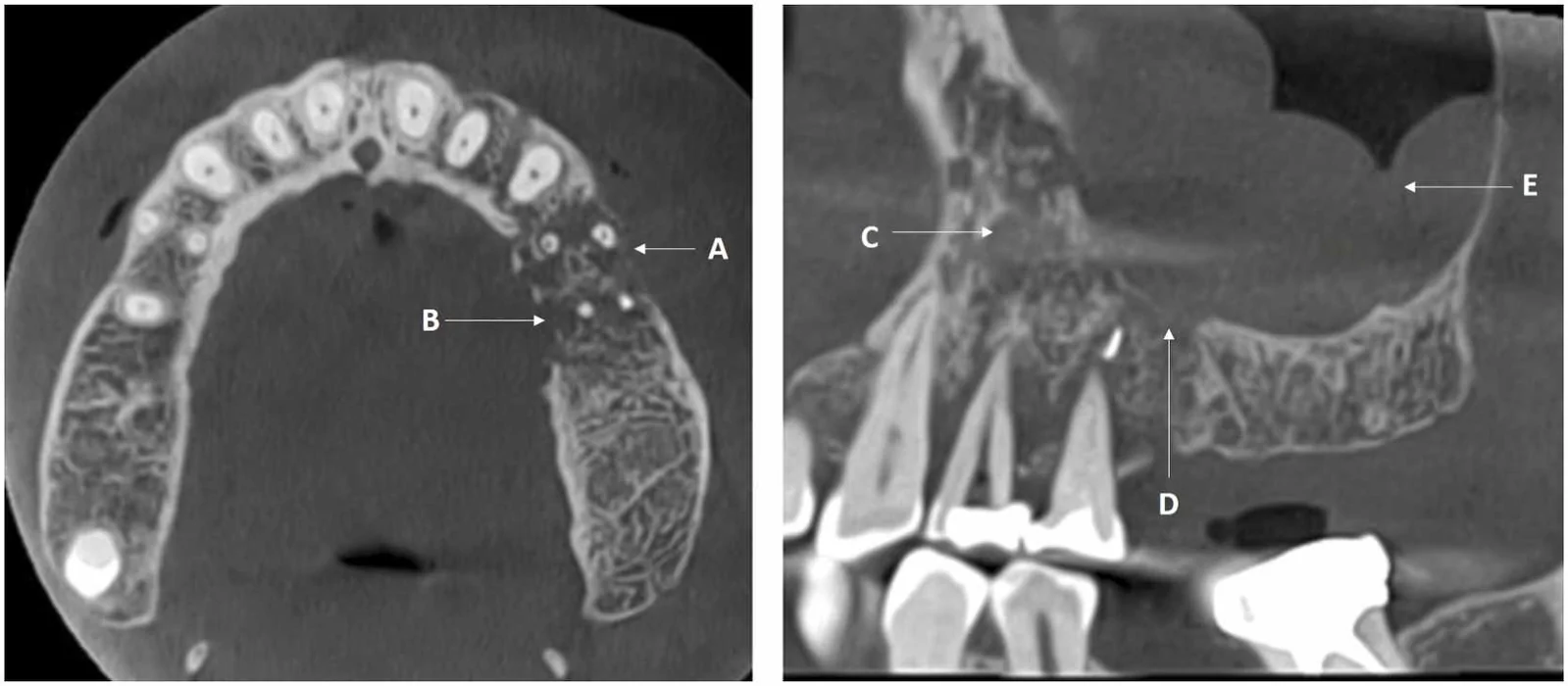
Axial CBCT section showing buccal cortical plate [A] and palatal cortical plate [B] perforation. Sagittal section demonstrating a moth-eaten osteolytic lesion [C] with maxillary sinus floor perforation [D] and thickening of the Schneiderian membrane [E].

By postoperative day 25, the patient developed nasal congestion. Clinical examination revealed Grade III mobility of teeth 24 and 25 and Grade II mobility of tooth 23. Cone-beam computed tomography (CBCT) revealed an ill-defined osteolytic lesion with a moth-eaten pattern of bone destruction involving the left posterior maxilla. The lesion perforated the floor of the maxillary sinus with extension into the sinus cavity and was associated with localized thickening of the schneiderian membrane. Perforations of both the buccal and palatal cortical plates were also evident (Fig. 2).

**Fig. 3:**
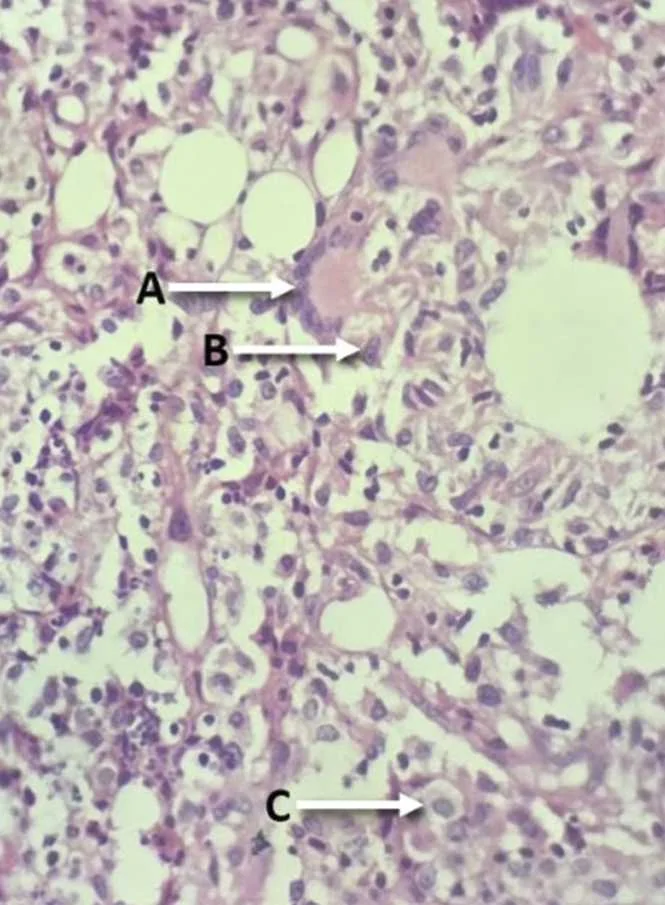
Histopathological section showing Langhans giant cells [A] with epithelioid cells [B] and macrophages [C].

Considering the progressive clinical deterioration and lack of response to endodontic retreatment and antibiotic therapy, teeth 24 and 25 were extracted, and extensive granulation tissue was curetted from the extraction sockets. Histopathological examination of the curetted specimen revealed Langhans giant cells within a cellular stroma exhibiting a dense mixed inflammatory infiltrate (Fig. 3), suggestive of a granulomatous infection.

Systemic evaluation revealed a normal chest radiograph, negative sputum acid-fast bacilli (AFB) examination, and a negative Mantoux test result. The patient had no known history of diabetes mellitus, HIV infection, immunosuppressive therapy, or previous TB or anti-tubercular treatment. There was no reported history of TB contact or constitutional symptoms, such as fever, night sweats, or weight loss. The patient was not pregnant at the time of presentation.

**Fig. 4:**
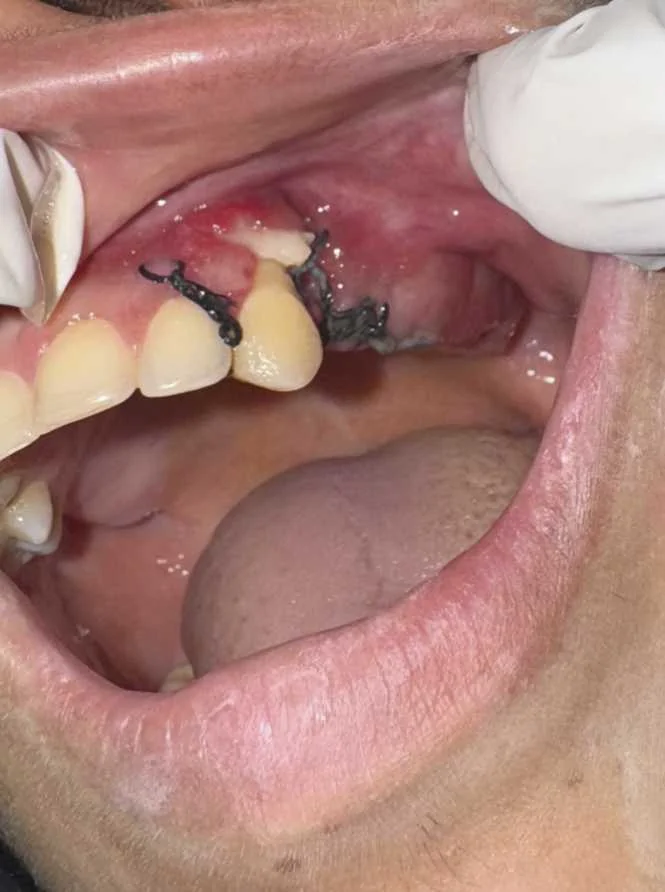
Sequestrum on the labial aspect of tooth 23.

One week after extraction, healing remained unsatisfactory, and a sequestrum was observed on the labial aspect of tooth 23 (Fig. 4). The patient subsequently underwent surgical debridement and sequestrum removal. Despite these negative screening results, cartridge-based nucleic acid amplification testing (CBNAAT) performed on the curetted bone tissue detected Mycobacterium tuberculosis without rifampicin resistance. In conjunction with the absence of pulmonary involvement, these findings established a diagnosis of primary extrapulmonary tuberculous osteomyelitis of the maxilla.

**Fig. 5:**
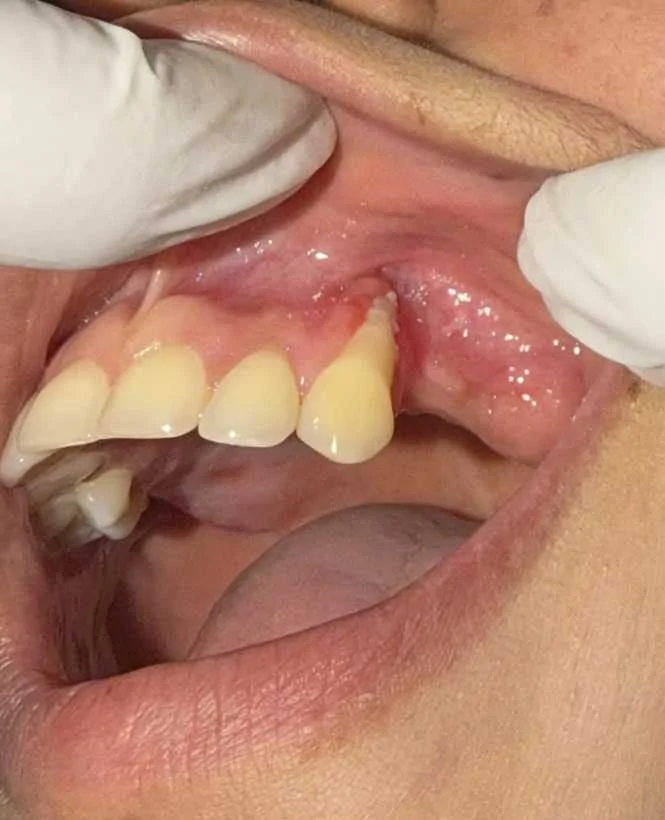
Clinical photograph demonstrating satisfactory mucosal healing following the initiation of anti-tubercular therapy.

Treatment with the National Tuberculosis Elimination Program (NTEP)-recommended drug-sensitive tuberculosis regimen of 2 months of isoniazid, rifampicin, pyrazinamide, and ethambutol (HRZE), followed by 4 months of isoniazid, rifampicin, and ethambutol (HRE) was initiated. The patient was maintained on regular follow-up and demonstrated progressive clinical improvement after the initiation of ATT. By the fifth week of therapy, satisfactory mucosal healing was achieved. Nasal congestion had completely resolved by the seventh week, and by the ninth week, the mobility of tooth 23 was markedly reduced (Fig. 5). The timeline of the progression of the disease is narrated in Table 1.

**Table 1:** Chronological clinical events and management

**Timeline**	**Clinical Events**
Day 0	Elective single-visit root canal treatment performed for teeth 24 and 25
Postoperative day 1	Severe postoperative pain developed
Postoperative day 19	Swelling with multiple purulent sinus openings on attached gingiva adjacent to tooth 24; root canal retreatment and empirical antibiotic therapy initiated without clinical improvement
Postoperative day 25	Nasal congestion developed; Grade III mobility of teeth 24 and 25 and Grade II mobility of tooth 23; CBCT demonstrated extensive osteolytic destruction of the left posterior maxilla; teeth 24 and 25 were extracted, and granulation tissue was curetted for biopsy
Histopathology report	Granulomatous inflammation with Langhans giant cells
1 week after the extraction of teeth	Persistent non-healing extraction site with sequestrum formation; systemic tuberculosis workup performed; curetted bone tissue submitted for CBNAAT
Following CBNAAT	Mycobacterium tuberculosis detected without rifampicin resistance; anti-tubercular therapy [ATT] initiated
5 weeks after ATT	Satisfactory mucosal healing
7 weeks after ATT	Complete resolution of nasal congestion
9 weeks after ATT	Marked reduction in tooth 23 mobility

## Discussion

TB remains a major global health challenge and continues to exhibit diverse extrapulmonary manifestations. Although pulmonary tuberculosis is the predominant clinical presentation, EPTB accounts for approximately 15–20% of all TB cases in immunocompetent individuals and a substantially higher proportion in immunocompromised patients [1]. TB involving the head and neck region is uncommon, accounting for only 2–6% of all TB cases. Tuberculous osteomyelitis constitutes less than 2% of skeletal TB cases, with maxillary involvement being exceedingly rare [2].

Primary oral tuberculosis without evidence of pulmonary disease is particularly unusual because the oral cavity possesses several inherent protective mechanisms against mycobacterial colonization and infection. The intact oral epithelium, cleansing action of saliva, salivary enzymes, local antibodies, and normal oral flora collectively provide resistance against infection. Disruption of these protective barriers by trauma, dental extraction, periodontal disease, or mucosal ulceration may facilitate the direct inoculation of Mycobacterium tuberculosis into the underlying tissues [3]. Following inoculation, microorganisms enter the nutrient blood vessels and induce the formation of microthrombi, which obstruct endosteal and periosteal circulation. This vascular compromise results in ischemia of the Haversian system, ultimately leading to the destruction of the corticomedullary architecture [4]. In the present case, the close temporal association with endodontic treatment raises the possibility that local tissue manipulation may have contributed to disruption of the local tissue barrier. A previous case report similarly proposed that M. tuberculosis present in the oral cavity might have entered periapical tissues through the root canal following endodontic treatment [5]. However, this association does not establish causality, and the exact route of infection in the present case remains uncertain.

Jaw involvement in tuberculosis may result from hematogenous dissemination from a latent focus, lymphatic spread, direct inoculation through damaged mucosa, or contiguous extension from adjacent structures, such as the maxillary sinus [6]. In the present case, the absence of pulmonary findings and negative systemic investigations favored a diagnosis of primary extrapulmonary tuberculosis of the maxilla. The later development of nasal congestion and imaging findings further suggested the extension of the inflammatory process towards the adjacent sinonasal region.

Maxillary involvement is considerably rarer than mandibular involvement because of the anatomical and vascular differences between the two jaws. The mandible contains a relatively dense cortical bone with limited collateral vascularity, predisposing it to chronic inflammatory conditions and sequestrum formation. In contrast, the maxilla is highly vascularized, with thin cortical plates, abundant cancellous bone, and efficient drainage, which collectively reduce the likelihood of persistent granulomatous infection. Furthermore, the relative paucity of medullary spaces within the maxilla limits the hematogenous localization of bacilli. These anatomical features likely account for the extremely low incidence of tuberculous osteomyelitis in the maxilla [3,7].

Clinically, tuberculous osteomyelitis of the jaws often presents with nonspecific features and may closely mimic conventional odontogenic infections [8]. In a systematic review of 301 oral and maxillofacial tuberculosis cases, de Farias Gabriel et al. reported intraosseous involvement in 25.9% of cases, with mandibular disease being substantially more common than maxillary involvement; only 11 cases (3.7%) affected the maxilla [9]. Reported manifestations of osseous involvement include pain, swelling, sinus tract formation, tooth mobility, delayed healing, sequestration, and osteolytic destruction [8,9]. The present case shares several of these features but is distinctive in its rapidly progressive course after endodontic treatment. The demographic profile also differed from that reported in the review, in which most cases occurred during the fifth and sixth decades of life, with a male predilection. The occurrence of maxillary osteomyelitis in a 38-year-old woman represents an uncommon demographic and anatomical presentation. Notably, 127 (51%) cases in the review were not associated with pulmonary manifestations, highlighting that oral tuberculosis may present as an isolated form of extrapulmonary disease [9]. Similarly, the present patient had no evidence of pulmonary involvement, and the maxillary lesion represented an isolated extrapulmonary manifestation of the disease.

Ulceration is the most frequently reported clinical presentation of oral tuberculosis [9]; however, no typical ulcer was observed in the present case. The lesion was initially considered a postoperative endodontic infection because it developed after root canal treatment and was associated with severe pain, swelling, and purulent sinus formation. However, the lack of response to endodontic retreatment, empirical antibiotic therapy, extraction of the involved teeth, and subsequent surgical curettage prompted the consideration of an atypical infectious process. This diagnostic challenge is consistent with the findings of de Farias Gabriel et al., in which tuberculosis was included among the initial clinical diagnostic considerations in only 11% of cases. In contrast, malignant lesions, particularly squamous cell carcinomas, are more frequently suspected [9].

Radiographically, tuberculous osteomyelitis of the jaws presents with variable and largely nonspecific features. The reported imaging findings include loss of normal trabecular architecture, ill-defined radiolucent areas, cortical bone destruction, and irregular osteolytic defects. Lesions may also exhibit a mixed radiolucent-radiopaque or “moth-eaten” appearance, occasionally accompanied by the formation of a sequestrum. Advanced lesions may demonstrate osteopenia, extensive osteolysis, reactive sclerosis, and periosteal bone formation [10]. In the present case, CBCT imaging revealed irregular osteolytic destruction of the maxillary alveolus with cortical disruption, findings that are consistent with chronic osteomyelitis. However, these radiographic features are not pathognomonic of this condition. They may overlap with a broad spectrum of infective, inflammatory, and neoplastic conditions, including actinomycosis, deep fungal infections, Langerhans cell histiocytosis, lymphoma, metastatic disease, and primary intraosseous malignancies.

The diagnosis of EPTB is often challenging because conventional investigations, such as sputum smear microscopy, chest radiography, and Mantoux testing, may be negative in isolated extrapulmonary lesions. Histopathological examination remains an essential diagnostic tool, particularly for atypical maxillofacial lesions. The presence of granulomatous inflammation with epithelioid histiocytes and Langhans giant cells strongly suggests TB, although similar findings may occasionally be encountered in fungal infections and sarcoidosis [11]. In the present case, Ziehl–Neelsen staining and systemic TB screening investigations were negative, underscoring the paucibacillary nature and diagnostic difficulty of isolated EPTB.

Molecular diagnostic techniques, such as cartridge-based nucleic acid amplification testing (CBNAAT), have substantially improved the diagnosis of extrapulmonary tuberculosis. CBNAAT enables the rapid detection of Mycobacterium tuberculosis DNA along with a simultaneous assessment of rifampicin resistance with high specificity [12]. In the present case, CBNAAT analysis of the curetted bone tissue established a definitive diagnosis despite negative routine screening investigations. These findings highlight the critical role of molecular diagnostics in persistent maxillofacial lesions that fail to respond to conventional therapy. A limitation of the present case was the absence of mycobacterial culture, which remains the definitive microbiological gold standard; however, the diagnosis was strongly supported by characteristic histopathological findings and positive CBNAAT results.

The management of tuberculous osteomyelitis of the jaws requires a combination of surgical and medical therapies. Surgical debridement aids in the removal of necrotic tissue, reduction of bacterial load, and procurement of tissue for histopathological and microbiological analyses. For drug-sensitive tuberculosis, the WHO regimen consists of 2 months of isoniazid, rifampicin, pyrazinamide, and ethambutol, followed by 4 months of isoniazid and rifampicin (2HRZE/4HR). Recently, a 4-month regimen comprising isoniazid, rifapentine, pyrazinamide, and moxifloxacin for 2 months, followed by isoniazid, rifapentine, and moxifloxacin for 2 months (2HPMZ/2HPM) has been recommended as an alternative for selected patients aged ≥ 12 years with drug-susceptible pulmonary tuberculosis. However, this shortened regimen is not recommended for the treatment of osteoarticular tuberculosis [13,14]. For the present case of skeletal tuberculosis, treatment was continued according to the Indian National Tuberculosis Elimination Program (NTEP) protocol with 2HRZE/4HRE. In this regimen, ethambutol is retained during the continuation phase, whereas pyrazinamide is discontinued. The NTEP protocol specifically recommends this regimen for drug-sensitive tuberculosis and provides for the extension of the continuation phase in skeletal tuberculosis when clinically indicated [15,16]. The patient showed progressive clinical and radiographic improvement during the follow-up period.

The pharmacological management of drug-resistant tuberculosis has evolved with the introduction of newer and repurposed agents, such as bedaquiline, pretomanid, delamanid, and linezolid, enabling shorter treatment regimens without injectable agents. Recent evidence supports six-month regimens, such as BPaLM (bedaquiline, pretomanid, linezolid, and moxifloxacin) and BDLLfx (bedaquiline, delamanid, linezolid, and levofloxacin), for eligible patients with MDR/RR-TB. These advances have shifted the management of drug-resistant tuberculosis towards shorter and safer regimens [14].

## Conclusion

This case is noteworthy because the lesion closely simulated a persistent post-endodontic infection and lacked systemic evidence of tuberculosis, thereby delaying the clinical suspicion of mycobacterial disease. This case illustrates the diagnostic difficulty associated with primary maxillary tuberculosis, which can mimic chronic odontogenic infection and osteomyelitis. Therefore, tuberculosis should be considered in the differential diagnosis of persistent, non-healing maxillary lesions that fail to respond to routine dental treatment, even in the absence of systemic manifestations. Early tissue biopsy and adjunctive molecular testing, particularly CBNAAT, are invaluable for establishing a timely diagnosis, facilitating appropriate therapy, and preventing unnecessary interventions.

## Author contributions

Ashir KR: Conceived the case report, established the clinical diagnosis, interpreted CBCT findings, and prepared the original manuscript. Manoj Vengal: Supervised the work and critically revised the manuscript for important intellectual content. Hamesh Gangadharan: Contributed to the clinical management and follow-up of the patient, participated in CBCT interpretation, and revised the manuscript. Rajeesh Mohammed P. K: Performed histopathological evaluation and contributed to manuscript revision. Vipindas A. P: Performed the surgical management, including extraction and debridement, and contributed to manuscript revision. Aswathi S: Contributed to the clinical management and follow-up of the patient, literature review and manuscript revision. All the authors have read and approved the final manuscript.

## CRediT authorship contribution statement

**Ashir KR:** Writing – review & editing, Writing – original draft, Investigation. **Manoj Vengal:** Writing – review & editing, Validation, Supervision. **Hamesh Gangadharan:** Writing – review & editing, Investigation. **P.K. Rajeesh Mohammed:** Writing – review & editing, Investigation. **A.P. Vipindas:** Writing – review & editing. **S. Aswathi:** Writing – review & editing.

## Patient consent

Written informed consent was obtained from the patient for the publication of this case report and clinical images. A copy of the consent form is available upon request.

## Ethical approval

Institutional ethics committee approval was not required for this case report.

## Declaration of Generative AI and AI-assisted technologies in the writing process

Generative artificial intelligence was used solely to improve the language, grammar, and readability of this manuscript. The authors have reviewed and approved the final version and take full responsibility for its content.

## Funding

No funding was received for this study.

## Declaration of Competing Interest

The authors declare that they have no known competing financial interests or personal relationships that could have appeared to influence the work reported in this paper.
